# Liver fat percent is associated with metabolic risk factors and the metabolic syndrome in a high-risk vascular cohort

**DOI:** 10.1186/1743-7075-7-50

**Published:** 2010-06-16

**Authors:** Michel R Hoenig, Gary Cowin, Raymond Buckley, Christine McHenery, Alan Coulthard

**Affiliations:** 1 Royal Brisbane and Women's Hospital, University of Queensland Centre for Clinical Research, Brisbane, Queensland, Australia; 2 University of Queensland, Brisbane, Queensland, Australia; 3 Department of Medical Imaging, Royal Brisbane and Women's Hospital and University of Queensland, Brisbane, Queensland, Australia

## Abstract

**Objective:**

To determine whether liver fat percent (LFP) is associated with the metabolic syndrome independently of visceral fat area (VFA).

**Methods:**

43 High-risk vascular patients not on lipid-lowering therapy were evaluated for the Adult Treatment Panel III (ATPIII) metabolic syndrome criteria and underwent magnetic resonance imaging (MRI) to quantify VFA and subcutaneous fat area (SFA) at the L4-L5 disc and liver magnetic resonance spectroscopy (MRS) to quantify LFP. Comparisons: 1. Baseline differences in patients with and without the metabolic syndrome 2. Forward binary logistic regression analysis of predictors of the metabolic syndrome with VFA, SFA and LFP as independents 3. Correlates of LFP.

**Results:**

43 patients were included in analysis. Patients with metabolic syndrome had greater VFA, SFA and LFP than patients without the metabolic syndrome (all p < 0.01). Of VFA, SFA and LFP, only LFP was associated with the diagnosis of the metabolic syndrome on forward binary logistic regression with an OR of 1.17 per 1% increase in LFP (p = 0.015). A 4% LFP threshold identified the metabolic syndrome with 84% sensitivity and 82% specificity. LFP correlated with waist circumference (r = 0.768), HDL-cholesterol (r = -0.342), triglyceride (r = 0.369), fasting glucose (r = 0.584) and the QUICK Index of insulin sensitivity (r = -0.679) (all p < 0.05)

**Conclusions:**

LFP is associated with the metabolic syndrome and renders the current gold standard of VFA redundant in this analysis. This measure of obesity-related cardiovascular risk requires further validation and evaluation in a prospective cohort.

## Background

In patients with established vascular disease, as in healthy patients, the diagnosis of the metabolic syndrome portends an increased risk of cardiovascular events and an increased risk of developing type 2 diabetes mellitus [1-4]. Large population studies utilizing computed tomography (CT) showed visceral fat area (VFA) at the umbilicus to be a superior determinant of metabolic risk factors and the metabolic syndrome than the subcutaneous fat area (SFA) after correction for body mass index (BMI) and waist circumference [5,6]. VFA is the 'gold standard' for quantifying obesity-related cardiovascular risk and has been independently linked to the development of coronary artery disease while SFA has not been shown to carry prognostic significance [7]. With the increasing use of magnetic resonance imaging (MRI) in lieu of CT for metabolic risk assessment, liver fat percent measured by magnetic resonance spectroscopy (MRS) has also emerged as a significant correlate of metabolic risk factors [8]. The potential utility of liver fat percent measured by MRS is highlighted by the finding of patients with 'metabolically-benign' obesity; i.e. obese patients with normal insulin sensitivity and lower liver fat percentages compared to insulin-resistant obese individuals [9]. This suggests that obese patients with low levels of liver fat may not have metabolic risk factors despite larger amounts of visceral fat compared to lean individuals. We hypothesized that liver fat percent (LFP) determined by MRS is associated with cardiometabolic risk factors independently and would render VFA redundant in predicting metabolic syndrome in a patients with vascular disease. Given the prognostic significance of the metabolic syndrome in patients with vascular disease, we evaluated the association of VFA, SFA and LFP with metabolic syndrome in a high-risk vascular cohort. We sought to identify a useful cut-off value of LFP which would identify patients with the metabolic syndrome, independently of other measures of obesity and the current gold standard measure of VFA.

## Methods and Procedures

### Study Population

Eligible patients for this study had coronary artery disease (CAD), ischemic stroke or CAD risk-equivalents. Eligible patients had to have at least one of the following: 1. CAD (positive angiogram or history of myocardial infarction) 2. peripheral vascular disease (ABI <0.9 or history of lower limb revascularization for atherosclerosis) 3. abdominal aortic aneurysm 4. carotid atherosclerosis with >50% narrowing 5. type II diabetes with age >50 and 3 additional risk factors (male sex, albuminuria, hypertension, HDL-C <40 mg/dl, TG >150 mg/dl, LDL-C >100 mg/dl, current smoking, diabetes duration >20 years) or 6. ischemic stroke. Study participants were in a clinically stable condition and were recruited from the vascular surgery outpatient department at the Royal Brisbane & Women's Hospital (RBWH). The study subjects were not taking lipid-lowering therapy at time of recruitment.

### Patient Data

Patient demographic information was collected including age, sex, qualifying criterion, self-reported race, current medications, alcohol intake, blood pressure, anti-hypertensive medication use, height, weight, waist and hip circumferences. Metabolic syndrome was defined as per the Adult Treatment Panel III (ATP III) criteria [10]. Fasting blood samples were analyzed for baseline lipids, glucose and insulin. Fasting lipid profile and glucose were determined using standard hospital methods. Insulins were measured by Chemilumnescent Immunoassay on a Beckman Coulter D × I800 as per the manufacturer's instructions. To determine insulin sensitivity, we used the Quantitative insulin sensitivity check index (QUICK Index) since this a superior linear correlate (r~0.8-0.9) of the reference standard glucose clamp than the Homeostasis model assessment (HOMA) model [11,12].

### MRI Measurement of Abdominal Fat Areas

MR imaging was performed with a Siemens Trio 3 T MRI system (Erlangen, Germany) using standard array coils with the subject supine. Breath-hold FISP images were centered on the L4-L5 intervertebral disc using standard localizer images with the following parameters: TR = 4 ms, TE = 2 ms, number of slices = 12, slice thickness = 8 mm, image matrix 256 × 256, field-of-view = 500 × 500 mm. The 4 slices that were best aligned with the L4-L5 disc (19, 20), were analyzed by a single operator (MRH) using the polygon region of interest in Escape Medical Viewer v3.2 to define visceral fat area (VFA) and subcutaneous fat area (SFA) as described previously [13]. Briefly, VFA and SFA were measured by fitting a spline curve to points on the border of the subcutaneous and visceral regions. Nonfat regions within the visceral region were also outlined with a spline fit and subtracted from the total visceral region.

### MRS measurement of Liver Fat Percent

Single voxel spin echo based PRESS spectra were used to measure liver fat using a Siemens Trio 3 T MRI system (Erlangen, Germany) using standard array coils with the subject supine as we have previously described [14,15]. Briefly, a voxel was positioned within the liver using standard localizer images, avoiding obvious vessels with the following parameters: TR = 2 sec, TE = 30 ms, voxel size 20 × 20 × 20 mm, 4 averages. The spectrum was acquired during a single breathhold. Spectra were processed using the standard Siemens software. Peak quantification was performed using the Siemens peak fitting package. The average liver fat content derived from the voxel was expressed as a percentage using the formula (CH_2 _+ CH_3_)/(H_2_O + CH_2 _+ CH_3_) ×100. We have previously correlated fat content determined with this protocol with intrahepatocellular lipid content on liver biopsy (r = 0.93) [15]. Liver fat percent measurements were highly reproducible with a coefficient of variation of 3.5%.

### Statistical Methods

The baseline characteristics of the included patients were summarized and the diagnosis of the metabolic syndrome as per the Adult Treatment Panel III (ATP III) criteria [10] was determined for each patient. We compared patients with and without the metabolic syndrome for various metabolic parameters and imaging parameters. We compared the means of continuous variables with a 2-tailed Student's t test for normally distributed variables, and with the Mann-Whitney U test for non-normally distributed variables. Categorical variables were analyzed with the chi-square test or Fisher exact test. We then used forward LR binary logistic regression to identify independent predictors of patients having the diagnosis of the metabolic syndrome. Candidate variables selected for logistic regression modeling were the magnetic resonance variables of adiposity VFA, SFA and LFP. Variables were only entered into the model if the p-value of the score statistic was less than the entry value of 0.05. Wald statistics and odds ratios were reported for variables in the final model and the overall model assessed the c-statistic for predicting the diagnosis of the metabolic syndrome. ROC curves were used to identify an optimal LFP associated with the metabolic syndrome with acceptable sensitivity and specificity. In order to determine correlates of LFP, we undertook univariate correlation with the metabolic syndrome criteria and insulin sensitivity (the QUICK Index) as independents and LFP as the dependent variable. The QUICK Index of insulin sensitivity for each subject with insulin and glucose data was calculated as 1/[log (fasting insulin, μU/ml) + log (fasting glucose, mg/dl)] [11]. Variables that correlated with LFP with a Spearman's p < 0.05 were subjected to stepwise multivariate linear regression and the R^2 ^change calculated with the addition of any variable to the model. To remove the influence of multicollinearity from the multiple regression model, variance-inflation factors (VIFs) were determined and variables with a VIF >4.0 were removed from the model. Residuals from the regression model were graphically examined. All analyses were done with statistics software (SPSS 16).

### Ethics Approval

This study is approved by the RBWH research ethics committee (2005/006A) and all study participants gave informed consent.

## Results

43 patients were enrolled in this MRI study. Baseline characteristics, including components of the metabolic syndrome, baseline lipid panel, insulin and QUICK Index of insulin sensitivity are shown in Table 1. The differences between the patients with and without the Metabolic Syndrome are summarized in Table 2 below and show the expected differences in various metabolic parameters. Patients with metabolic syndrome had greater amounts of VFA, SFA and LFP compared to patients without the metabolic syndrome (p < 0.01). In order to determine which of these MRI or MRS-derived parameters is most strongly associated with the metabolic syndrome, we subjected the outcome of metabolic syndrome to binary logistic regression analysis with VFA, SFA and LFP as independents. On logistic regression, only liver fat remained in the model with no contribution from VFA or SFA; beta 0.15, Wald's statistic 5.9, OR 1.17, p = 0.015. Thus, the current gold standard for assessing obesity-related cardiovascular risk is redundant when LFP is considered as a covariate. Each increase in LFP by 1% is associated with an odds ratio for metabolic syndrome of 1.17. The overall c-statistic for LFP in identifying the metabolic syndrome was 0.92 (p < 0.001) and in our sample a liver fat percent of >4.0% identified the metabolic syndrome with 84% sensitivity and 82% specificity.

**Table 1:** Patient Characteristics of Patients Included in the Analysis

	Mean ± SD or n (%)
Age	70 ± 8
Males	35 (81)
Females	8 (19)
Race	
White	42 (98)
Non-White	1 (2)
Height (cm)	172 ± 10
Weight (kg)	79 ± 19
Body Mass Index	26 ± 5
Diabetic, n (%)	10 (23)
Metabolic Syndrome (ATPIII), n (%)	22 (53)
Waist (cm)	101 ± 16
Fasting Glucose (mg/dl)	112 ± 40
HDL Cholesterol (mg/dl)	44 ± 17
Triglycerides (mg/dl)	142 ± 74
Systolic Blood Pressure (mmHg)	142 ± 19
Diastolic Blood Pressure (mmHg)	72 ± 9
Average Number of ATP III Criteria	2.7 ± 1.5
Daily Intake of Alcohol (standard drinks) Fasting Insulin (mU/L)	1.4 ± 1.8 7 ± 4
QUICK Index	.25 ± .03
Visceral Fat Area (cm^2^)	203 ± 111
Subcutaneous Fat Area (cm^2^)	240 ± 113
Liver fat Percentage	6.6 ± 6.3
Qualifying Criterion^a^	
Coronary Artery Disease	10 (24)
Peripheral Vascular Disease	23 (55)
Carotid Atherosclerosis >50%	12 (29)
Abdominal Aortic Aneurysm	10 (24)
Ischemic Stroke	8 (19)
Diabetes	10 (24)

^a^Patients frequently had >1 inclusion criterion

**Table 2:** Characteristics of the Patients with and Without the Metabolic Syndrome

Variable	Mean ± SD or n (% of Patients)		P value MetSyn vs Without MetSyn
		
	With MetSyn, n = 23	Without MetSyn, n = 20	
Number of ATPIII Criteria	3.9 ± 0.7	1.3 ± 0.7	<0.001

Waist (cm)	110 ± 10	91 ± 15	<0.001

HDL-C (mg/dl)	39 ± 18	50 ± 15	0.007

TG (mg/dl)	177 ± 81	102 ± 38	<0.001

Fasting Glucose (mg/dl)	127 ± 50	96 ± 8	0.001

Hypertension, n (%)	17 (74)	10 (50)	0.065

Diabetic, n (%)	10 (43)	0 (0)	0.001

Male, n (%)	19 (83)	16 (80)	0.83

Age	69 ± 7	69 ± 10	0.75

Body Mass Index	29 ± 4	23 ± 3	<0.001

Percentage Liver fat	10.4 ± 6.5	2.4 ± 2.1	<0.001

Visceral Fat Area (cm^2^)	263 ± 113	134 ± 53	<0.001

Subcutaneous Fat Area (cm^2^)	282 ± 116	191 ± 91	0.007

Fasting Insulin (mU/L)	9.3 ± 4.9	4.9 ± 2.7	0.001

QUICK Index of Insulin sensitivity	0.242 ± 0.031	0.263 ± 0.26	0.001

Despite the criticism of the label of metabolic syndrome as being no greater than a sum of risk factors, it remains a clinically-useful label denoting an increased cardiovascular risk. However, given reports of metabolically-benign obesity [9] and the belief that insulin resistance drives the increased risk of cardiovascular events in obese individuals [16], we assessed the utility of LFP >4% in identifying insulin-resistant patients. We compared the QUICK Index of insulin sensitivity in patients with an LFP >4% and <4% and found that patients with LFP >4% were significantly more insulin resistant (lower QUICK Index score) than those with an LFP <4% p = 0.001 indicating that LFP >4% can identify an insulin-resistant population. Next, we sought to confirm the association between LFP and metabolic indicators of risk by correlating the LFP to each of the metabolic syndrome criteria and the QUICK Index. The QUICK Index and all the metabolic syndrome criteria, except for the hypertension criterion, were significant correlates of liver fat percent as shown in Table 3. On stepwise multivariate analysis of the significant univariate correlates as independents and LFP as the dependent variable, only waist circumference is retained in the model with an R^2 ^of 0.6, p < 0.001. Hence, waist circumference is an important determinant of LFP but does not explain all the variation in LFP. There was no correlation of LFP with the number of standard alcoholic beverages consumed daily by the study participants (r = -0.156, p = 0.364).

**Table 3:** Univariate Correlates of Liver Fat Percent

	Waist Circumference	HDL Cholesterol	Fasting Triglyceride	Fasting Glucose	ATP III Hypertension Criterion	QUICK Index
Spearman's coefficient	.768	-.342	.369	.584	.139	-.679

p-value	<.001	.041	.027	<.001	.418	<0.001

## Discussion

We have shown that liver fat percent is associated with the metabolic syndrome independently of visceral fat area in high-risk vascular patients with each percent increase in liver fat percentage being associated with an odds ratio of 1.17 of having the metabolic syndrome. This is the first head-to-head comparison of these two obesity-related cardiovascular risk measures and in our cohort, visceral fat area was a redundant predictor of the metabolic syndrome. A cut-off of 4% liver fat identified patients with the metabolic syndrome with an 84% sensitivity and 82% specificity. Patients with >4% liver fat had significantly lower QUICK Insulin sensitivity indices than patients with <4% liver fat which is consistent with this threshold being able to identify insulin-resistant patients. Liver fat percent correlates with insulin sensitivity and all components of the metabolic syndrome except for hypertension. On multivariate regression, waist circumference is the only significant determinant of percent liver fat (R^2 ^of 0.6, p < 0.001).

The association of LFP with the metabolic syndrome is a clinically-significant one since the metabolic syndrome in patients with established vascular disease identifies a cohort at an increased risk of cardiovascular events [1-4]. Indeed, liver fat percent has been shown to be greater in obese insulin-resistant patients vs obese insulin-sensitive patients (10.5% vs 5.6%) with the obese insulin-sensitive patients having a carotid intima-media thickness comparable to healthy normal weight individuals [9]. Hence, while the definition of obesity has evolved from weight to body mass index and more recently waist circumference and subsequently visceral fat area, liver fat percent may represent the future determinant of obesity-related cardiovascular risk assessment. Indeed, it is possible that obese patient with higher amount of visceral fat area but low liver fat percent may have a cardiovascular event rate comparable to normal-weight individuals. While our data are encouraging in showing that liver fat percent may better identify the at-risk patient than visceral fat area, our data set is small and cross-sectional. A large prospective cohort is required to determine if liver fat percent is independently associated with cardiovascular events. One limitation of this test is the availability of MRI; however, from a pragmatic perspective, such a measure would be preferable to visceral fat area since liver fat percent is easier to measure and does not require tracing of fat areas on abdominal MRI slices. Further, it would be valuable to validate liver fat percent as a measure of obesity-related cardiovascular risk in various ethnic groups since Asians, in particular, are prone to cardiovascular disease and diabetes at lower waist circumference or body mass index than Europids [17,18]. In summary, we have shown liver fat percent to be associated with the metabolic syndrome independently of visceral fat area. Our results suggest that this measure requires further validation as a maker of obesity-related cardiovascular risk and assessment in prospective cohort studies.

## Competing interests

The authors declare that they have no competing interests.

## Authors' contributions

MRH conceived of the study, participated in its design and coordination and extracted and analyzed data. GC provided the imaging protocols, assisted in acquiring images and extracted & interpreted data. RB and CM assisted in modifying imaging protocols and image acquisition and storage. AC provided oversight for image acquisition and participated in study coordination. All authors read and approved the final manuscript.
